# Severe Osteogenesis Imperfecta in Cyclophilin B–Deficient Mice

**DOI:** 10.1371/journal.pgen.1000750

**Published:** 2009-12-04

**Authors:** Jae Won Choi, Shari L. Sutor, Lonn Lindquist, Glenda L. Evans, Benjamin J. Madden, H. Robert Bergen, Theresa E. Hefferan, Michael J. Yaszemski, Richard J. Bram

**Affiliations:** 1 Department of Immunology, Mayo Clinic College of Medicine, Rochester, Minnesota, United States of America; 2 Department of Transplant Biology, Mayo Clinic College of Medicine, Rochester, Minnesota, United States of America; 3 Department of Pediatric and Adolescent Medicine, Mayo Clinic College of Medicine, Rochester, Minnesota, United States of America; 4 Department of Orthopedics Research, Mayo Clinic College of Medicine, Rochester, Minnesota, United States of America; 5 Mayo Proteomics Research Center, Mayo Clinic College of Medicine, Rochester, Minnesota, United States of America; University of Washington, United States of America

## Abstract

Osteogenesis Imperfecta (OI) is a human syndrome characterized by exquisitely fragile bones due to osteoporosis. The majority of autosomal dominant OI cases result from point or splice site mutations in the type I collagen genes, which are thought to lead to aberrant osteoid within developing bones. OI also occurs in humans with homozygous mutations in Prolyl-3-Hydroxylase-1 (LEPRE1). Although P3H1 is known to hydroxylate a single residue (pro-986) in type I collagen chains, it is unclear how this modification acts to facilitate collagen fibril formation. P3H1 exists in a complex with CRTAP and the peptidyl-prolyl isomerase cyclophilin B (CypB), encoded by the *Ppib* gene. Mutations in CRTAP cause OI in mice and humans, through an unknown mechanism, while the role of CypB in this complex has been a complete mystery. To study the role of mammalian CypB, we generated mice lacking this protein. Early in life, *Ppib*-/- mice developed kyphosis and severe osteoporosis. Collagen fibrils in *Ppib*-/- mice had abnormal morphology, further consistent with an OI phenotype. *In vitro* studies revealed that in CypB–deficient fibroblasts, procollagen did not localize properly to the golgi. We found that levels of P3H1 were substantially reduced in *Ppib*-/- cells, while CRTAP was unaffected by loss of CypB. Conversely, knockdown of either P3H1 or CRTAP did not affect cellular levels of CypB, but prevented its interaction with collagen *in vitro*. Furthermore, knockdown of CRTAP also caused depletion of cellular P3H1. Consistent with these changes, post translational prolyl-3-hydroxylation of type I collagen by P3H1 was essentially absent in CypB–deficient cells and tissues from CypB–knockout mice. These data provide significant new mechanistic insight into the pathophysiology of OI and reveal how the members of the P3H1/CRTAP/CypB complex interact to direct proper formation of collagen and bone.

## Author Summary

Osteogenesis Imperfecta (OI), also known as “brittle bone disease,” is an inherited condition with multiple defects in collagen-containing structures, including the bones, skin, and other connective tissues. Patients with OI suffer from short stature, scoliosis, thin skin, hearing loss, and, most notably, fragile bones that break with little or no trauma. Although many cases are due to dominantly inherited point mutations in the collagen genes, autosomal recessive forms have been described due to defects in the genes for Prolyl-3-Hydroxylase-1 (LEPRE1) and Cartilage-Associated Protein (CRTAP), proteins that modify newly synthesized procollagen. Some patients with OI do not have mutations in any of the known disease-related genes. Here, through the use of newly generated knockout mice, we identify the endoplasmic-reticulum resident prolyl-isomerase cyclophilin B (CypB) as a new autosomal recessive OI gene in mice. CypB, P3H1, and CRTAP were shown to have interrelated effects in maintaining their respective protein levels and ability to bind to collagen. These studies enhance our understanding about how collagen, the most abundant protein in the body, becomes properly assembled to form bones with adequate strength.

## Introduction

OI is an inherited disorder of collagen, affecting one in 12,000 newborns [1],[2]. Point mutations in the type I collagen genes COL1A1 or COL1A2 are responsible for the majority of cases, which are typically autosomal dominant. The severity of symptoms of OI is highly heterogeneous, ranging from mild increased risk of bone fractures to neonatal lethality. Recently, mutations in the endoplasmic reticulum (ER)-resident proteins Prolyl-3-hydroxylase-1 (P3H1, also known as LEPRE1) or its binding partner CRTAP were found to be causative in a subset of autosomal recessive forms of OI in humans and/or mice [3]–[5]. P3H1 hydroxylates a single residue (proline-986) of procollagen shortly after secretion into the ER, and this modification is postulated to facilitate the proper folding or stability of collagen trimers [6]. Although CRTAP mutants also show deficient prolyl-3-hydroxylation of collagen, how this protein supports the function of P3H1 has not been elucidated. Cyclophilin B has been found in association with P3H1 and CRTAP, however its function in the complex is completely unknown [5],[7],[8].

Cyclophilins form a class of proteins originally discovered by virtue of their high specific affinity for the immunosuppressant drug cyclosporin A [9]. They are thought to accelerate the folding of proteins by catalyzing the isomerization of peptidyl-proline bonds, which are normally constrained in their rotation [10]. Cyclophilins are highly conserved throughout evolution, and the different isoforms display substantial sequence similarity in their central cyclosporine-binding domains. Individual differences at N- and C-termini of the various family members direct their subcellular localizations and protein-protein interactions. The initial cyclophilin discovered, cyclophilin A, is primarily cytosolic, and was shown to mediate the immunosuppressive effect of cyclosporin by creating an inhibitory complex with calcineurin [11],[12]. Cyclophilin A plays an essential role in blocking the activity of the lymphocyte tyrosine kinase Itk, and mice lacking the *Ppia* gene develop allergic symptoms due to enhanced Itk-induced T_H_2 cells [13].

Cyclophilin B (CypB) is a highly related family member that is present within the endoplasmic reticulum (ER) of all cell types [14]. In vitro studies have previously suggested a possible role for CypB in multiple diverse functions, including immunosuppression [15], chemotaxis [16], hepatitis C virus replication [17], and prolactin signaling [18]. As an ER-resident, it was also postulated to be involved in post-translational folding of secreted proteins, although a requirement for this property has not been clearly established to date. CypB has also been found to associate with collagen [19].

An important feature of patients with OI is the high degree of variability in the severity of their disease symptoms. The causes for this variability are not understood, however it is likely that there are unlinked modifier genes that impact the phenotypic spectrum of disease severity [2],[20]. In addition, some patients with OI do not have mutations in any known disease-related genes. Thus, it is important to identify additional genes that direct the proper biosynthesis and assembly of procollagen into fibrils. In this report, we identify CypB as a new OI phenotype disease-gene in mice, and explore the function of its interactions with P3H1 and CRTAP.

## Results

### Reduced body size and kyphosis in CypB knockout mice

**Figure 1:**
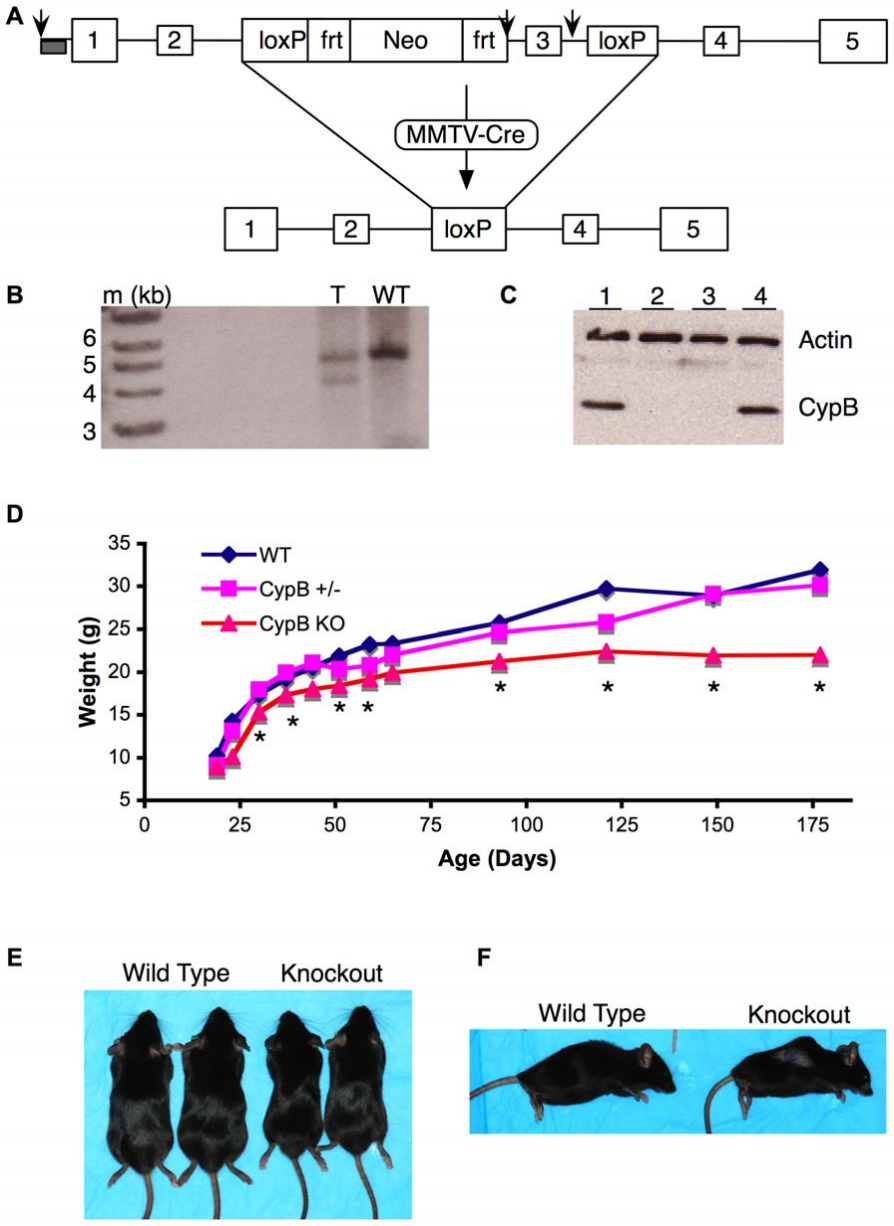
Reduced body size in CypB–knockout mice. (A) The third exon of the *Ppib* gene, encoding CypB, was targeted by loxP sites knocked-in to either side. Arrows indicate SacI sites used for Southern blot analysis of the targeted clones, probed from outside of vector sequences (grey rectangle). Mice bearing this allele were mated to MMTV-Cre transgenic mice to delete the exon in the germ cells of female pups. Their offspring demonstrated complete loss of CypB. (B) Southern blot of genomic DNA from targeted (‘T’) and wild-type (‘WT’) ES clones. (C) Western blot of CypB in thymocytes from wild type (lanes 1, 4) or knockout (lanes 2, 3) mice. (D) Average weights of wild-type, heterozygous, or homozygous CypB–knockout mice. n = 3 WT littermates; n = 10 *Ppib+/−*; n = 12 *Ppib−/−* mice. Asterisks indicate points that were statistically significantly different by one-tailed students T-test (p<0.05) from the heterozygote weights. (E, F) Typical appearance of wild type and knockout mice.

To determine the role of CypB in vivo, we targeted exon 3 by homologous recombination, and generated mice bearing the knockout allele (Figure 1A). The resulting allele contains an out of frame join between exons 2 and 4, thus was predicted to completely inactivate the gene. Mating of heterozygote mutant mice gave rise to viable homozygous knockouts at the expected Mendelian ratio. Successful deletion of the gene was verified by Southern blotting (Figure 1B) and by Western blotting (Figure 1C, Figure S1A). mRNA generated from the mutated allele accumulated to significantly lower levels than normal, most likely due to nonsense-mediated decay (Figure S1B).

**Figure 2:**
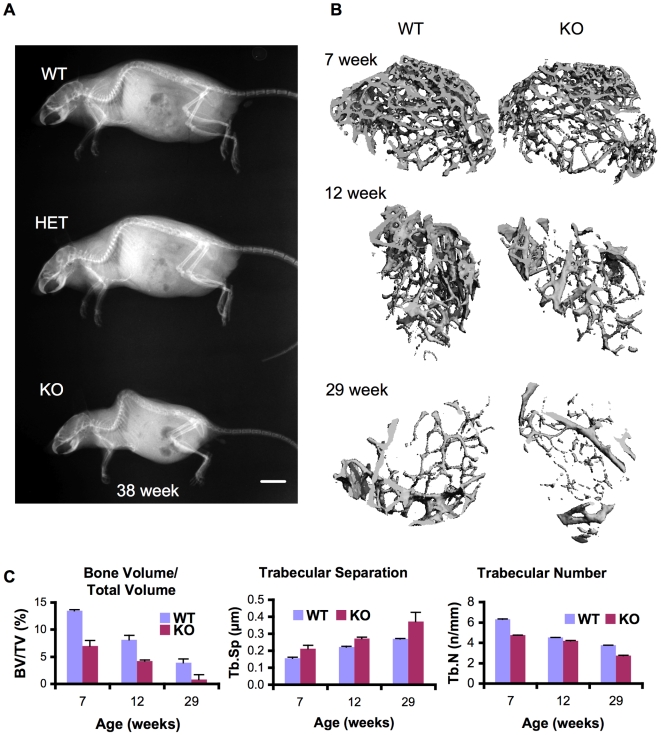
Abnormal bone in CypB–knockout mice. (A) Total body radiographs of 38-week-old mice. Note the enhanced curvature of the spine in the *Ppib−/−* mouse. Bar = 1 cm. (B) 3D reconstruction of slices from micro CT analysis of femurs from CypB knockout and littermate control mice. (C) Bone volume/total volume, trabecular separation, and trabecular number in femurs analyzed by microCT at the indicated ages.

Because CypB is expressed in all cell types, and is highly conserved from yeast to humans, we anticipated that homozygous loss might cause developmental abnormalities during embryogenesis. Surprisingly, CypB knockout mice appeared normal at birth, and both sexes were fertile. In addition, *Ppib^−/−^* mothers had no apparent difficulties giving birth, feeding, or raising their pups. On the other hand, homozygous CypB knockout mice had reduced body size and weight, in comparison to littermate controls (Figure 1C and 1D), and typically died between 40 and 50 weeks of age of unclear etiology. A striking feature was pronounced kyphosis, noted as early as 8 weeks after birth that progressed in severity with age (Figure 1F, Figure 2A, Figure S2A). Dual-energy x-ray absorptiometry further demonstrated this kyphosis, and suggested that knockout mice had reduced bone density (data not shown). Although rhizomelia has been described in some types of osteogenesis imperfecta [4],[5], we did not observe differences in the ratio of femur to tibia lengths in CypB-deficient mice compared to littermate controls (0.819±0.027 vs. 0.826±0.013) (Figure S2B).

### Severe osteopenia and aberrant type I and type II collagen in the absence of CypB

To study the pathophysiology of altered bone development, *Ppib^−/−^* or littermate control mice were euthanized and femurs dissected for analysis by microcomputed tomography. Serial sections of femurs revealed dramatically reduced amounts of trabecular bone in mice lacking CypB (Figure 2B). Average bone volume was significantly reduced and the separation between trabeculae was increased in mutant mice (Figure 2C).

**Figure 3:**
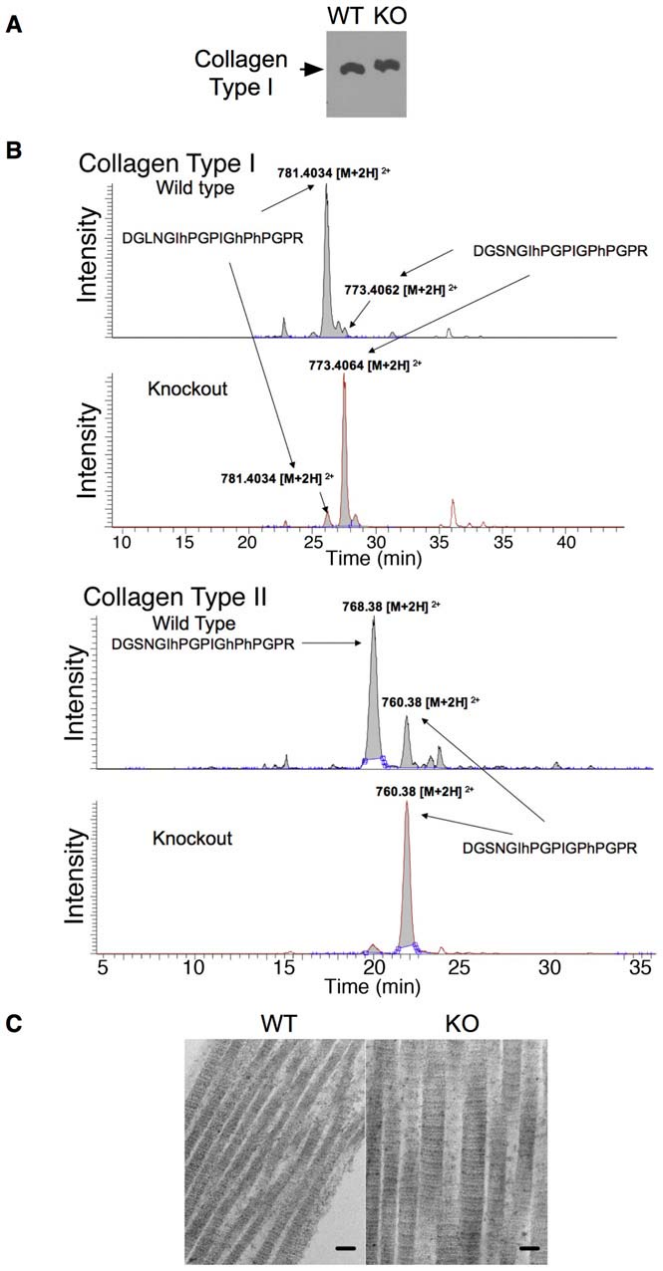
Altered collagen in CypB–knockout mice. (A) Skin fibroblasts from wild-type or CypB knockout mice were cultured in serum free medium. Secreted collagen from the supernatant was electropheresed on 5% SDS-PAGE and detected by Western Blotting. Type I collagen from mutant animals demonstrated a subtle but reproducible decrease in migration compared to wild type. (B) Mass-spectroscopic analysis of type I and type II collagen from bone and cartilage. Chromatogram of the parent ions in the mass spectra from wild type and *Ppib−/−* collagen tryptic-peptides. Wild-type collagen is 4-hydroxylated at pro-981 and pro-987, and 3-hydroxylated at pro-986. Collagen from *Ppib−/−* mice primarily lacked the pro-986 hydroxylation. Indicated peaks were analyzed by MS2 and MS3 to identify hydroxylated proline residues (‘hP’). (C) Transmission electron micrographs of subcutaneous tissue revealing abnormally thick collagen fibrils in mutant mice in comparison to control mice. (bar = 100 nm).

Because of the critical role for collagen in directing bone formation [21], we next analyzed collagen from *Ppib^−/−^* mice, as was performed for CRTAP knockout mice [5]. Skin fibroblasts were prepared from knockout and littermate control embryos and maintained in culture. Collagen secreted into serum-free medium was collected and fractionated on SDS-PAGE. Western blotting revealed slightly delayed migration (Figure 3A), consistent with increased post-translational modification, as was noted previously in collagen from LEPRE1 or CRTAP mutant cells [3],[4].

Samples of bone and cartilage were extracted by limited pepsin digestion, and proteins resolved by SDS-PAGE. Coomassie blue stained bands were excised, subjected to trypsinization, and analyzed by tandem mass spectrometry. The tryptic peptide containing proline-986 (975-DGLNGLPGPIGPPGPR-990) was identified, and further analyzed by MS2 and MS3 for hydroxylation modifications (Figure 3B, Figure S3). Remarkably similar to collagen from LEPRE1 mutant humans and CRTAP knockout mice [5], multiple analyses demonstrated an almost complete absence of 3-hydroxy proline in the peptide at Pro-986 from CypB-knockouts, while collagen from wild type and heterozygote littermates had abundant amounts of peptide containing hydroxy-proline in this position. The known sites of prolyl–4 hydroxylation at residues 981 and 987 were appropriately modified by hydroxylation in the peptides from both wild type and knockout collagen. Similar results were obtained for Type II collagen from cartilage (Figure 3B).

**Figure 4:**
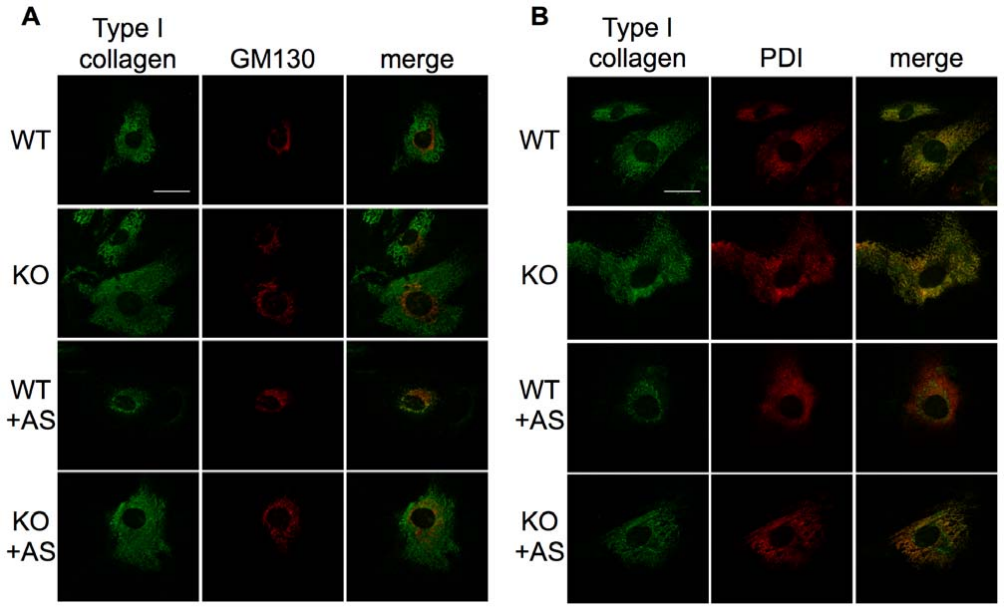
Abnormal localization of collagen in CypB–deficient cells. MEFs prepared from wild-type (WT) or CypB knockout mice (KO) were cultured with or without 24hr stimulation with ascorbic acid (+AS). Cells were fixed, permeabilized, and co-stained for intracellular collagen and the Golgi marker GM130 (A) or the ER-resident protein PDI (B). (bar = 40 µm).

3-hydroxylation of Pro-986 in type I collagen is required for correct fibril formation because mutation of P3H1 or CRTAP causes the accumulation of aberrant fibrils with wider than normal diameter [4],[5]. We therefore examined subcutaneous collagen fibrils in CypB knockout mice by transmission electron microscopy. The majority of collagen fibrils in these mice were on average 1.45 times wider than similar samples from littermate control mice (114.6 +/− 22.4 nm vs. 78.6 +/− 12.4 nm diameter) (Figure 3C). Because of the reported interaction between CypB and type I collagen precursors during their transit through the export pathway [19], we next stained cells for endogenous collagen. In the absence of ascorbic acid, the majority of collagen co-localized with the ER-resident marker PDI (Figure 4). Upon addition of ascorbic acid for 24 hours, collagen in wild type cells concentrated in the area of the golgi apparatus, as indicated by co-staining with a GM130 antibody [22]. However, in cells lacking CypB, a significant amount of collagen remained in the ER (Figure 4). Thus, CypB is important for appropriate subcellular localization of collagen within the protein secretion system.

### Abnormal skin phenotype in CypB deficient mice

**Figure 5:**
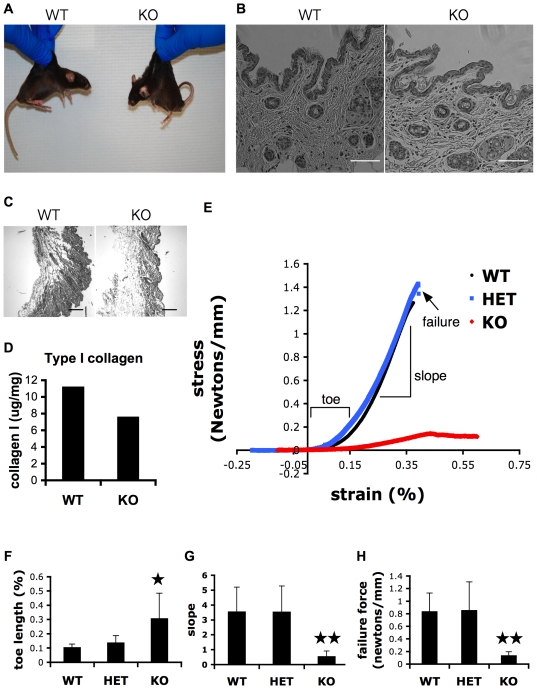
Abnormal skin in CypB–deficient mice. (A) Typical appearance of wild-type (left) and *Ppib−/−* (right) mice demonstrating the laxity of skin. (B) Sections of skin were fixed and stained with H&E, revealing normal cellular architecture of the dermis. Bar = 200 µm. (C) Sirrius red staining demonstrates reduced collagen in skin sections from CypB-knockout mice. Bar = 250 µm. (D) Quantitation of total type I collagen from skin determined by ELISA. (E) Representative stress vs. strain (SvS) curves of skin from wild type, heterozygote, or CypB knockout mice. (F) Toe lengths of SvS curves indicating the normal range of skin stretchiness. (G) Slopes of SvS curves prior to fracture indicate that CypB−/− collagen fibers are more easily pulled past each other. (H) Force of breakage was much lower in CypB-deficient skin (For all comparison, n = 4; *P<0.05; **P<0.01).

Thin skin is a common feature of patients with OI. We noticed that CypB −/− mice were easily identified by their loose skin (Figure 5A). To determine whether this was related to a defect in collagen, sections of skin were fixed and stained by H & E, or Sirrius Red (Figure 5B and 5C). Cellular appearance was normal, however we noted decreased intensity of Sirrius red staining, suggesting lower concentrations of collagen in CypB-deficient skin. Total collagen was extracted from skin samples with pepsin digestion, and tested by ELISA for type I collagen content, verifying this result (Figure 5D). To quantitate the defect further, we performed tensile strength testing of skin samples, as was done previously for Tenascin-X deficient mice, which are a model for Ehlers−Danlos syndrome [23]. Comparison of stress versus strain (Figure 5E) revealed several abnormal properties of CypB−/− skin samples, which were very similar to published results from Tenascin-X knockout mice. The ‘toe’ region of the stress-strain curves, representing normal ranges of skin stretching, were much longer than normal (Figure 5F). The steep linear portion of the curves had flatter slopes (Figure 5G), demonstrating dramatically lower stiffness of the aligned collagen fibers, thought to be due to the density and cross-linking of the fibrils [23]. Lastly, much lower forces were required to break CypB-knockout mouse skin (Figure 5H). All three parameters were essentially the same between wild type and heterozygote mice.

Lastly, we prepared collagen from skin samples, and analyzed it by mass spectroscopy. As described for bone and cartilage collagen, there was a severe loss of triple-hydroxylated collagen representing the 986-hydroxylated peptide (Figure S4).

### CypB regulates P3H1

**Figure 6:**
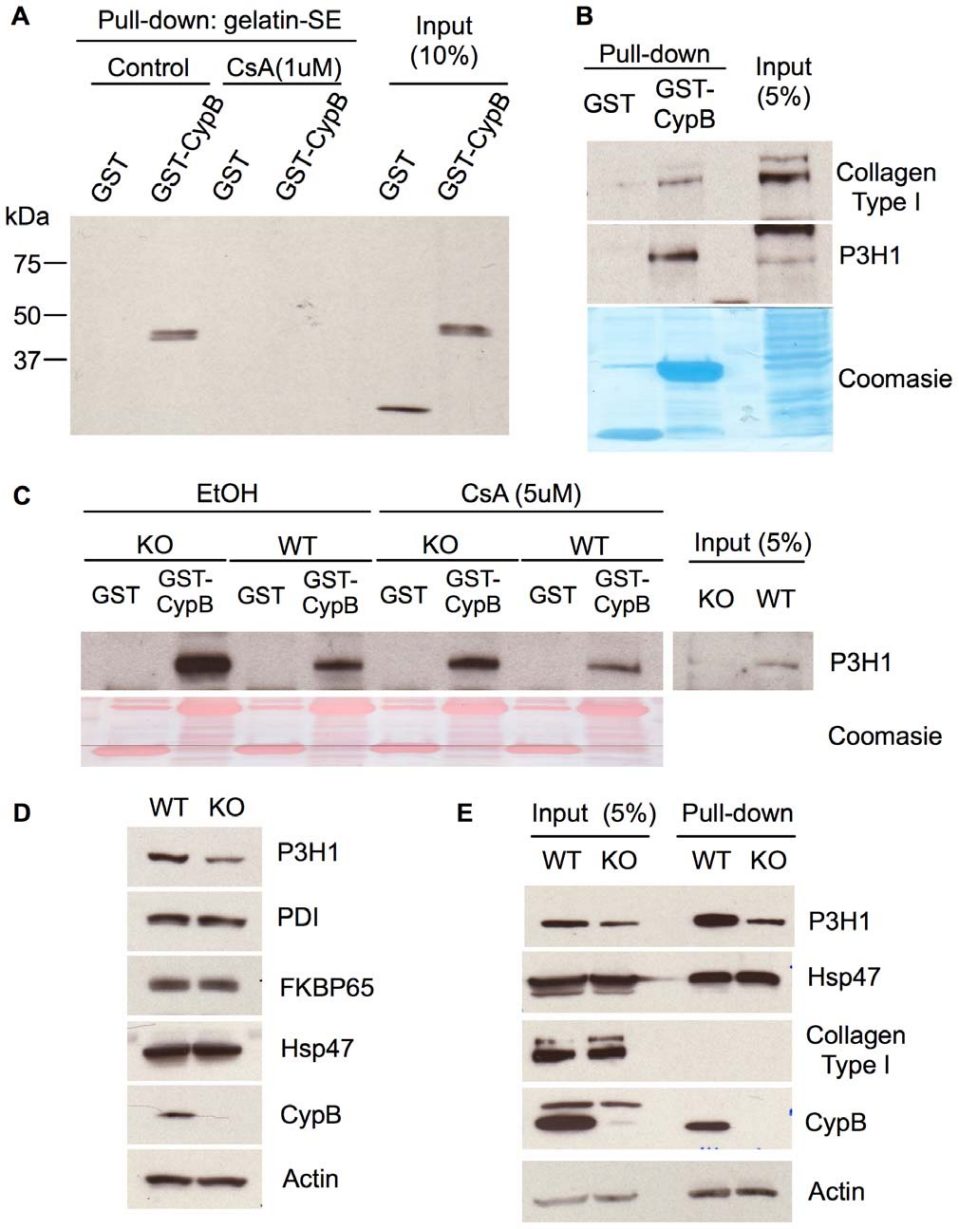
Decreased levels of P3H1 protein in the absence of CypB. (A) GST-cyclophilin B or GST was mixed and pulled down with gelatin sepharose in the absence or presence of cyclosporine A. Proteins were visualized by blotting with antibody to GST. (B) Lysates from wild type cells were mixed with recombinant GST-cyclophilin B or GST, and bound proteins isolated using GSH-agarose prior to SDS PAGE. Proteins were visualized by western blotting with antibodies to the indicated proteins. (C) Binding of GST-CypB to P3H1 in lysates from wild-type (WT) or CypB-knockout fibroblasts (KO) was detected by chromatography on glutathione-agarose followed by western blotting for P3H1. Although P3H1 is present at much lower levels in the absence of CypB, a greater fraction of it binds to recombinant CypB in vitro. (D) Western blot of the indicated proteins from lysates of wild-type and CypB knockout skin fibroblasts. Blotting for beta-actin was performed as a loading control. (E) Proteins from lysates of wild-type and CypB–knockout skin fibroblasts were isolated on gelatin-sepharose. Bound proteins were eluted and detected by western blotting with antibodies to the indicated proteins.

Several other investigators have reported that CypB is able to bind to collagen, and to the P3H1 / CRTAP complex [5],[8]. To verify this in our system, we prepared recombinant CypB as a GST-fusion protein from E.coli, and tested its ability to bind to collagen in the form of gelatin linked to sepharose. GST-CypB indeed bound stably to gelatin-sepharose, and there was no interaction between the control protein GST and gelatin (Figure 6A). Addition of cyclosporine A (CsA) completely blocked the CypB-gelatin interaction, indicating that the enzymatic active site of CypB (which binds to the twisted proline intermediate during the cis-trans peptidyl prolyl isomerase reaction [24]) is important for affinity to collagen.

We investigated whether recombinant CypB would also bind to P3H1 from cells. Lysates from wild type fibroblasts were prepared, incubated with the fusion protein, and then passed over glutathione-agarose resin to recover GST-CypB. We observed a moderate degree of retention of P3H1 specifically to GST-CypB, and none to GST alone, indicating they may indeed interact. (Figure 6B). However, we noted that Type I collagen also was retained specifically in these pull-down experiments, raising the alternative possibility that P3H1 might be indirectly associating with CypB through collagen monomers acting as intermediate bridges. To further explore this latter possibility, we repeated the pull-down experiments in the presence of CsA, to inhibit CypB binding to collagen. However, CsA had only a modest inhibitory effect on the CypB-P3H1 association (Figure 6C), suggesting that the proline-binding site of CypB is probably not involved in interaction with P3H1. Interestingly, GST-CypB pulled down a much larger fraction of cellular P3H1 when added to lysates from *Ppib−/−* cells, consistent with the idea that under normal conditions, most P3H1 is already bound to endogenous CypB (Figure 6C).

In conducting these studies, we also found that the amount of P3H1 was relatively reduced in CypB-deficient cells (Figure 6D). This effect was specific because there was not a similar reduction in other ER-resident proteins, such as PDI, FKBP65, HSP47. However, the residual P3H1 retained its ability to bind to collagen, as determined by affinity to gelatin-sepharose (Figure 6E). Thus, stable accumulation of P3H1 in fibroblasts depends upon CypB, however it does not require CypB in order to associate with collagen.

### P3H1 regulates CypB

**Figure 7:**
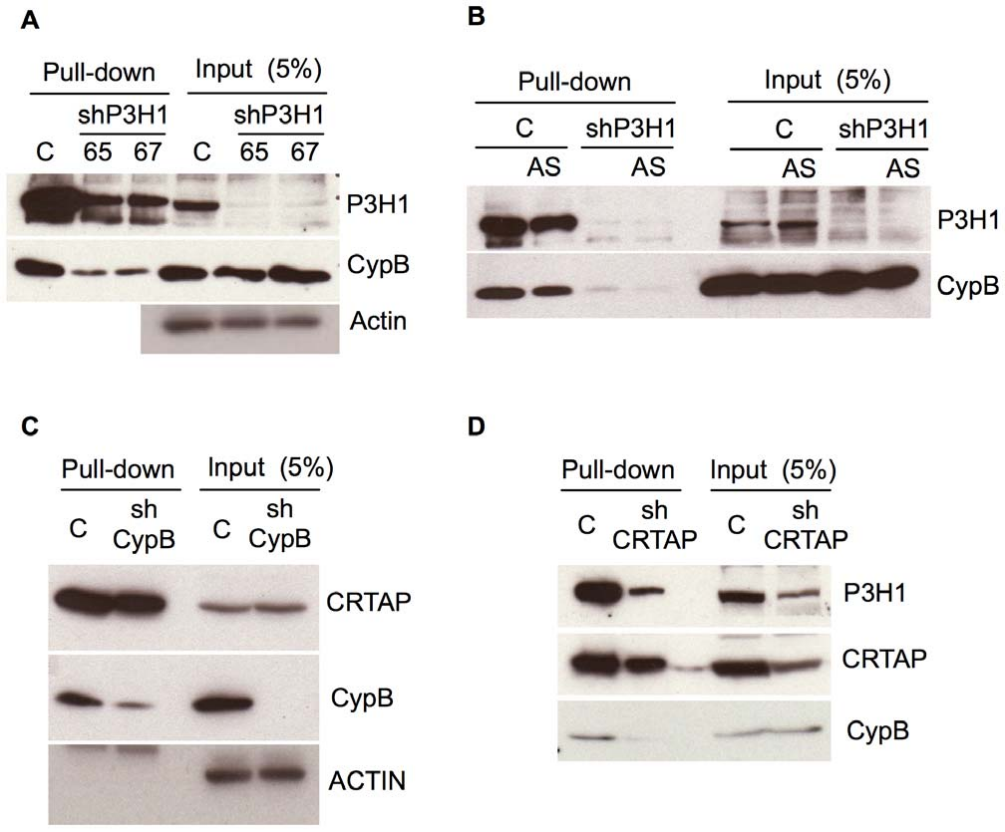
P3H1 and CRTAP are required for efficient binding of CypB to collagen in vitro. (A) Mouse fibroblasts were transduced with two different shRNA lentiviruses (#65 and #67) to knockdown P3H1 or a control lentivirus (“C”). Lysates were mixed with gelatin-sepharose to adsorb collagen binding proteins, and then recovered for western blotting, as indicated. Total cellular levels of CypB were not affected by knockdown of P3H1, however binding to gelatin was reduced. (B) As described in (A) in the absence or presence (“AS”) of ascorbic acid in the medium. (C) HeLa cells were transduced with a CypB specific shRNA lentivirus or control and lysates probed for gelatin-binding proteins as described. Knockdown of CypB did not affect CRTAP levels or binding to gelatin. (D) Knockdown of CRTAP in HeLa cells reduced P3H1 cellular levels and blocked the binding of CypB to gelatin in vitro.

To investigate the reciprocal relationship of P3H1 effects on CypB, several commercial shRNA lentivirus preparations were tested, and two were found to cause significant depletion of P3H1 protein in murine fibroblasts. We found that in both cases, reduction of P3H1 did not have any effect on intracellular levels of CypB (Figure 7A). On the other hand, knockdown of P3H1 severely reduced the amount of CypB that could bind to gelatin-sepharose in vitro, indicating that a substantial portion of CypB-gelatin binding depends upon the P3H1 protein. This result was obtained regardless of whether the cells were grown in the presence or absence of ascorbic acid (Figure 7B), which is known to increase collagen production and hydroxylation [25],[26].

### CRTAP regulates P3H1 and CypB

Like P3H1, CRTAP has been reported to be mutated in mice and humans with OI. CRTAP does not have a known enzymatic function, and, although collagen from CRTAP−/− mice was shown to have reduced prolyl–3-hydroxylation, its mechanism in this process is not understood. Unfortunately, we were unable to obtain antibodies that recognized the murine CRTAP, thus we turned to a human cell system to explore its potential interactions. HeLa cells were found to have easily detectable CRTAP. CypB was knocked down using a shRNA lentivirus, and lysates were tested as described above. Although CypB was efficiently reduced, there was no impairment of either CRTAP accumulation, nor of the binding of CRTAP to gelatin-sepharose (Figure 7C).

Knockdown of CRTAP in HeLa cells, on the other hand, caused a substantial depletion of P3H1 (Figure 7D). In addition, although there was not an effect of loss of CRTAP on CypB intracellular levels, we did observe a dramatic impairment in the ability of CypB from these lysates to bind to gelatin-sepharose (Figure 7D). We conclude that CRTAP maintains the appropriate cellular accumulation of P3H1, and facilitates CypB binding to collagen.

## Discussion

Collagen makes up the most abundant protein in the body, and is critically important for structural elements of skin and subcutaneous tissue, as well as for providing the osteoid framework on which calcium phosphate precipitates during bone formation. The problem of how microscopic cells synthesize and properly build structures, such as bones, that are many orders of magnitude larger than themselves is an important one in biology and medicine, however the details of this complex process are incompletely understood. It has been of great value to study human and mouse mutants with abnormal bone development, like OI, in order to identify the critical components of this system.

Eight types of OI have been described [2]. The initial classification of types I – IV were based primarily on clinical presentation, with type II (typically lethal in the perinatal period) being the most severe. All four have subsequently been shown to result from mutations in COL1A1 or COL1A2 genes. Types VII and VIII are autosomal recessive, and are due to mutations in CRTAP and LEPRE1 respectively, while the genetic basis of type V and VI remains unknown. We provide here the first evidence indicating an essential role for CypB in the biosynthesis of bone in mice. Many of the features of OI were recapitulated in *Ppib−/−* mice, including reduced body mass, kyphosis, and reduced bone density. In addition to the poor quality and quantity of bone mineralization, we found increased laxity and decreased strength of the skin in these animals. Along with the abnormal appearance of collagen fibrils by ultrastructural analysis of subcutaneous tissue, this suggests that CypB provides an important function during the generation of collagen in more tissues than just the skeletal system. The occurrence of kyphosis in these mice may therefore be a function of both decreased strength of connective tissue as well as abnormal vertebral bones, since several genetic conditions with primarily soft-tissue manifestations are associated with spinal deformities [27]–[29]. Although this is the first report of deletion of CypB and its role in bone formation, it is intriguing to note that CypB has been linked previously to a skin disorder in horses known as HERDA, in which affected animals develop hyperextensible skin and scarring along the back [30]. HERDA is an autosomal recessive disorder, and is due to a 39G>R point mutation in CypB, however the molecular mechanism was not previously described [31]. We suspect it may be related to the mechanism we propose in this report, although why it selectively affects the skin in horses is not clear. However, a distinguishing feature of these two genetic conditions is the ultrastructural appearance of collagen fibers, which are reduced in diameter in HERDA-affected horses and larger than normal in CypB and CRTAP mutant mice. This difference may be due to a residual altered chaperone activity or glycosaminoglycan-binding property of mutated CypB in HERDA [31], which is not present in the knockout mice.

Cyclophilins form an ancient, highly conserved group of proteins that are present in all eukaryotic cells, and in some bacteria [32]. The role of CypB has been a mystery, although it has been suggested to mediate prolactin receptor and Interferon Regulatory Transcription factor 3 (IRF-3) signal transduction [18],[33],[34]. Given the high degree of conservation of this gene, it was surprising to us that *Ppib−/−* mice were viable. However, we did observe increased rates of death at approximately 1 year of age. Our preliminary results seem to indicate that providing food and water at a low level in the cages as mice age improves their overall survival.

Our molecular studies provide a framework for understanding the relatively complex interrelationships of the members of the P3H1/CRTAP/CypB complex. Intracellular levels of P3H1 were dependent upon both CypB and CRTAP individually. However, P3H1 did not require either partner in order to maintain its ability to bind stably to collagen in the form of gelatin-sepharose. Others have previously shown that the prolyl-3-hydroxylation reaction of P3H1 in the absence of partner proteins is intact [8]. However, we did not observe appropriate prolyl–3-hydroxylation of residue pro-986 in collagen from CypB-deficient cells. This is likely due, in part, to reduced P3H1 levels in these mutant cells.

**Figure 8:**
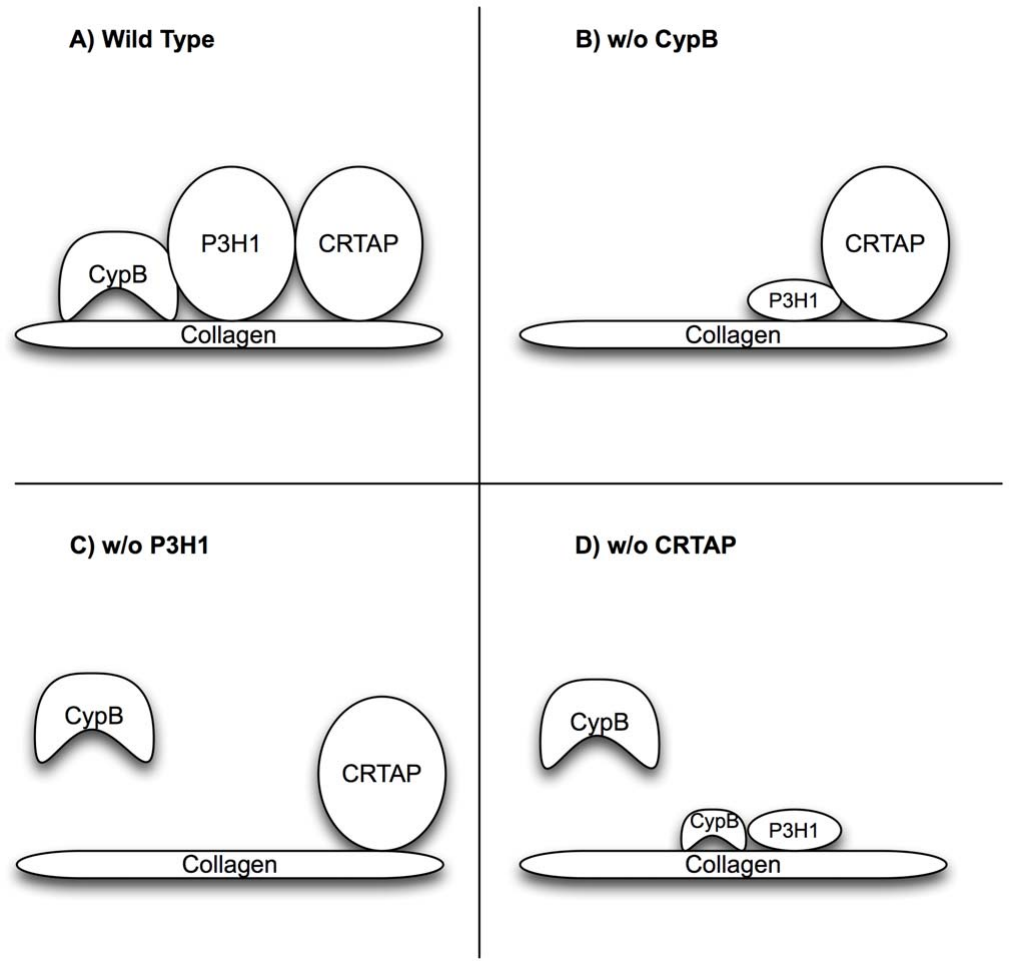
A working model to summarize the interaction data posits that CRTAP and CypB both serve to stabilize the accumulation of P3H1 within cells. P3H1 not only hydroxylates the 3 position of proline 986, but also plays a key role by facilitating the binding of CypB to collagen within the ER. In all 3 conditions that lead to OI, P3H1 hydroxylation is absent, and CypB binding to collagen is reduced or absent.

On the other hand, although intracellular levels of CypB did not depend upon either CRTAP or P3H1, knockdown of either significantly prevented the binding of CypB to gelatin-sepharose. Although CypB has an endogenous collagen-binding property, we observed that the extent of binding is relatively low. CypB in complex with P3H1 was a much better substrate for affinity to gelatin-sepharose. One conclusion from these studies is the observation that in each condition that can cause OI (i.e. loss of any member of the P3H1/CRTAP/CypB complex), there is a significant loss of CypB binding to collagen (Figure 7). A possible model, therefore (Figure 8), is that an important role for P3H1 is to stabilize CypB on the newly synthesized procollagen molecule, in order to facilitate its prolyl-isomerization. Proline makes up almost 20% of the residues in type I collagens, so it would not be surprising if these proteins needed special help during folding. CRTAP, according to this model, would provide support by maintaining P3H1 levels. Consistent with this model is the recent finding that the cis-trans peptidyl prolyl isomerase activity of CypB is available and active in the complex with P3H1 and CRTAP [7]. This is in agreement with our finding that CypB/P3H1 binding is not ablated by addition of CsA.

Alternative mechanisms for CypB are possible, however. Others have shown that abnormally folded collagen is degraded by proteasomes, which reside in the cytosol, thus would require retrotranslocation [35]. Cyclophilin B has been implicated in retrotranslocation [18], thus its absence in knockout mice likely compounds the problem in cells with high collagen synthetic rates. Our finding of abnormally localized collagen in the ER of CypB deficient cells is consistent with a delay in their processing or removal to the cytosol for proteasomal degradation.

As noted above, there are some OI patients that do not have identified disease mutations. Our results suggested to us the possibility that CypB might be a potential underlying cause in a subset of these people. Indeed, while this manuscript was in revision, several humans with recessive OI of the severe neonatal type were reported to have mutations in the *PPIB* gene [36],[37]. The clinical features of the human cases were very similar to findings presented here, although they had increased numbers of bone fractures, possibly due to the relatively greater stress of birth in humans compared to mice. In addition, diseases of other types of collagen, such as Ehlers-Danlos syndrome [38], may also result from alterations in CypB.

Cyclosporin A is a commonly used immunosuppressant. It works by binding to cyclophilin A, which then blocks the interaction of calcineurin with its downstream target NFAT [12],[39]. It is known, however, that cyclosporine also binds cyclophilin B, and causes it to be released from the cell [40]. Cyclosporin A has multiple toxic side effects in humans, including reduction in bone mineral density [41]. Although the effects of cyclosporin A are complex, this toxicity may in part be mediated through inhibition of CypB, as suggested by this new work. Our findings also raise the possibility that this could turn out to be a side effect of non-immunosuppressive cyclophilin binding drugs, which are currently being tested clinically for anti-viral properties [42] and proposed as a new treatment for muscular dystrophy in order to inhibit cyclophilin D [43].

## Materials and Methods

### Mice

All animal procedures were reviewed and approved by the Institutional Animal Care and Use Committee. A targeting plasmid was generated using a BAC clone containing the entire *Ppib* gene from 129SvJ genomic DNA. Germline transmission in 129 x C56B6 mice was achieved essentially as previously described [44]. The initial mice carried a conditional knockout allele (Figure 1) in case total deletion would be embryonic lethal. Mice were crossed to MMTV-Cre transgenic mice to cause deletion of the floxed exon 3 in female germ cells, after which the Cre transgene was bred out of the offspring, since it was no longer needed. Pups from these females were heterozygous for the KO allele, and were bred as needed to generate heterozygote or homozygote mutant, or littermate control wild type mice.

### Cell culture and lentiviral knockdown

Mouse embryonic fibroblasts (MEFs) or skin fibroblasts were generated as previously described [45]. Cells were maintained in DMEM +10% fetal calf serum with 50 µg/ml ascorbate (sigma) unless otherwise indicated. For isolation of secreted collagen, cells were cultured in serum free DMEM containing ascorbate. For knockdown in skin fibroblasts, Lentiviral vectors encoding small hairpin interfering RNA sequences (shRNA) were obtained from sigma (LEPRE1 and CypB) or Open Biosystems (CRTAP). Lentiviral vectors were transfected in 293 FT cells with packaging plasmids (Invitrogen) for generating lentiviral particles. Skin fibroblasts were transduced with specific lentiviruses and selected by 2 ug/ml puromycin (Invitrogen).

### Radiographs and cancellous bone analysis

Mice were euthanized and analyzed by radiography (GE AMX 4) of whole body or hind limbs. The femur was cleaned of soft tissue and measured ex-vivo on a uCT 35 micro-CT scanner (Scanco Medical, Basserdorf,Switzerland). Trabecular architecture was measured distally from the growth plate (70 kVP, increment 7 um) at a resolution of 7 um. The analysis region was represented by 100 slices.

### Collagen analysis

To analyze modification of secreted procollagen, confluent fibroblasts were stimulated in serum-free conditions containing ascorbate during the 48 hrs. The harvested media were electrophoresed on 5% SDS polyacrylamide gels under reducing conditions and processed for westernblotting with type I collagen antibody (SouthernBioTech). To examine the overall architecture and quantify the collagen content in a given area of mouse skin tissue, sections of paraffin-embedded skin sample from abdomen were stained with H&E or Picrosirius red (Polysciences, Inc.). Type I collagen in the extract from skin samples was quantified with Mouse Type I Collagen Detection Kit (Chondrex, Inc.)

### Protein identification via in-gel trypsin digest and nanoLC-MS/MS with hybrid orbitrap/linear ion trap mass spectrometry

To determine prolyl 3-hydroxylation in collagen samples from skin, bone and cartilage, collagen was solubilized and extracted by a limited digestion with pepsin (100 µg/ml; Calbiochem) in 0.5N acetic acid for 48 hr. This extracts were resolved by SDS-PAGE. For collagen samples obtained from skin fibroblast culture, the harvested culture media were concentrated by Centricon (Millipore) and resolved by SDS-PAGE. The Coomassie Blue stained SDS-PAGE gel bands were prepared for mass spectrometry analysis using the following procedures. The gel bands were destained with 50 mM Tris, pH 8.1/50% acetonitrile until nearly clear. The bands were then reduced with 30 mM DTT/50 mM Tris, pH 8.1 at 55°C for 40 minutes and alkylated with 40 mM iodoacetamide at room temperature for 40 minutes in the dark. Proteins were digested in-situ with 30 ul (0.004 ug/ul) trypsin (Promega Corporation, Madison WI) in 20 mM Tris pH 8.1/.0002% Zwittergent 3–16, at 37°C overnight followed by peptide extraction with 60 ul of 2% trifluoroacetic acid, then 60 ul of acetonitrile. The pooled extracts were concentrated to less than 5 ul on a SpeedVac spinning concentrator (Savant Intruments, Holbrook NY) and then brought up in 0.15% formic acid/0.05% trifluoroacetic acid for protein identification by nano-flow liquid chromatography electrospray tandem mass spectrometry (nanoLC-ESI-MS/MS) using a ThermoFinnigan LTQ Orbitrap Hybrid Mass Spectrometer (ThermoElectron Bremen, Germany) coupled to an Eksigent nanoLC-2D HPLC system (Eksigent, Dublin, CA). The digest peptide mixture was loaded onto a 250nl OPTI-PAK trap (Optimize Technologies, Oregon City, OR) custom packed with Michrom Magic C8 solid phase (Michrom Bioresources, Auburn, CA). Chromatography was performed using 0.2% formic acid in both the A solvent (98%water/2%acetonitrile) and B solvent (80% acetonitrile/10% isopropanol/10% water), and a 5%B to 45%B gradient over 60 minutes at 400 nl/min through a Michrom packed tip capillary Magic C18 75 µm x 200 mm column. The initial LTQ Orbitrap mass spectrometer experiment was set to perform a FT full scan from 375–1600 m/z with resolution set at 60,000 (at 400 m/z), followed by linear ion trap MS/MS scans on the top five ions. Dynamic exclusion was set to 2 and selected ions were placed on an exclusion list for 40 seconds. The lock-mass option was enabled for the FT full scans using the ambient air polydimethylcyclosiloxane (PCM) ion of m/z = 445.120024 or a common phthalate ion m/z = 391.284286 for real time internal calibration.

The experiment to target the hydroxyproline sites of the peptide DGLNGLPGPIGPPGPR, relied on performing MS3 on the abundant y-ion from fragmentation N-terminal to a proline residue. The Orbitrap full scan used a list of masses for the [M+2H]2+ ions of this peptide without and with 1 to 5 hydroxyprolines to trigger the data dependant ion trap MS/MS scans. The ion trap MS/MS/MS scans were triggered on the most abundant ion of the MS/MS scan. If no masses from the list were detected in the Orbitrap full scan, then the most abundant ions triggered the MS/MS events.

The MS/MS raw data were converted to DTA files using extract_msn.exe from Bioworks 3.2 and correlated to theoretical fragmentation patterns of tryptic peptide sequences from the Swissprot databases using Mascot™ 2 (Matrix Sciences London, UK). All searches were conducted with fixed modification of carbamidomethyl-cysteine and variable modifications allowing oxidation of methionines for methione sulphoxide, oxidation of prolines for hydroxyproline, and protein N-terminal acetylation. The search was restricted to full trypsin generated peptides allowing for 2 missed cleavages and was left open to all species. Peptide mass search tolerances were set to 10 ppm and fragment mass tolerances are set to±0.8 Daltons. All protein identifications were considered when Mascot individual peptide scores were above the 95% percentile for probability and rank number one of all the hits for the respective MS/MS spectra. The MS/MS/MS scans were analyzed manually.

### Transmission electron microscopy

Skin tissues taken from lower abdomen were processed for transmission electron microscopy as described [46]. Skin biopsy was fixed overnight in 0.1% glutaraldehyde and 4% formaldehyde in 0.1-M phosphate buffer at pH 7.2. After primary fixation, tissues were rinsed in 0.1-M phosphate buffer, followed by postfixation in phosphate-buffered 1% osmium tetroxide. After rinsing in three changes of distilled water, the tissue was stained en bloc with 2% uranyl acetate at 55°C. The tissues were rinsed in distilled water, dehydrated in progressive concentrations of ethanol and propylene oxide, and embedded in Spurr resin. The resin was polymerized at 65°C. Thin sections were mounted on copper grids for evaluation with a transmission electron microscope (JEOL JEM-1400; JEOL USA, Peabody, Massachusetts). To analyze fibril morphology and density, we selected 5 adjacent regions lying just beneath the epidermal basement membrane from each of 4 sex- and age- matched wild type and CypB knockout littermates.

### Immunofluorescence

MEFs were cultured on coverslides, fixed with 2.5% paraformaldehyde in PBS and permeabilized using 0.2% Triton X-100 in PBS for 2 min. Cells were blocked with PBS containing 5% goat serum and stained for intracellular collagen with anti-mouse collagen antibody (AB765P; Chemicon). Cellular localization was verified by costaining with anti-PDI (stressgen) or anti-GM130 antibody (BD). Samples were visualized using a Zeiss epifluorescence microscope.

### Cell lysis, immunoblotting, and affinity chromatography with gelatin-sepharose

Cells were lysed in 1% NP-40, 20 mM HEPES [pH 7.4], 5 mM NaCl, 5 mM MgCl2, 1 mM EGTA, 1 mM EDTA, 1 mM PMSF, 10 µg/ml leupeptin, and 45 µg/ml aprotinin. 50 µg of protein lysates were resolved by 8% SDS-PAGE and transferred to charged nylon membranes (Millipore). Western blots were probed for cyclophilin B with rabbit polyclonal antibody (Affinity BioReagents) or mouse monoclonal antibody (k2E2; Abcam), type I collagen (SouthernBioTech), P3H1 (Abnova), PDI (Stressgen), Hsp47 (Stressgen), FKBP65 (BD), GST (Sigma), CRTAP (Abnova) and beta-actin (Sigma). Reactive bands were visualized with a secondary antibody conjugated with HRP (Zymed) and chemiluminescence (Thermo scientific). For pulldowns, cell extracts were mixed with GST-cyclophilin B or GST and treated with GSH-agarose (GE healthcare), washed in lysis buffer and eluted by SDS sample buffer. For binding assays with gelatin-sepharose (GE healthcare), lysates containing approximately 1 mg of total protein were mixed with gelatin-sepharose for 3 h at 4°C with continuous rotation. The resulting precipitate was washed twice with lysis buffer, subjected to SDS-PAGE, and then blotted for the indicated protein.

### Quantitative RT–PCR

Total RNA was purified with TRIZOL (Invitrogen) from splenocytes or MEFs and used as a template for synthesis of cDNA with SuperScript III First Strand RT-PCR kit (Invitrogen). With TaqMAN Gene Expression Assays (Applied Biosystems), *Ppib* and Actin mRNA levels were measured on a ABI 7900HT Thermocycler (Applied Biosystems). All q-PCR reactions were run in quadriplicate. *Ppib* level were normalized to constitutively expressed actin. The graph of fold change was generated by ΔΔCt values.

### Biomechanical testing

Skin from 38-weeks old female mice was biomechanically tested for tensile strength as described [23]. Stress was calculated by normalizing measured force with cross-sectional area. The strain at a stress equivalent to 0.05 (Newtons/mm) was used as the toe length. Slope was calculated with data in the linear portion (above 0.2 Newtons/mm). Highest force was normalized with cross-sectional area for failure force.

## Supporting Information

Figure S1 (A) Western blot of lysates from splenocytes (left) and MEFs (right) using two different antibodies to verify absence of immunoreactive CypB in knockout cells. (B) Real time rtPCR of CypB mRNA normalized to actin message levels in splenocytes from wildtype or knockout animals.
(1.19 MB TIF)

Figure S2 Radiographs. (A) Total body radiographs showing the skeletons of mice at 24 and 63 weeks of age. Bar = 1 cm. (B) Radiographs of lower limbs. The measured ratios of femur to tibia were similar in all mice, regardless of CypB.
(1.86 MB TIF)

Figure S3 MS2 and MS3 analysis of peptides from bone, demonstrating the data used for identification of hydroxylated proline residues.
(1.95 MB TIF)

Figure S4 Ion-current LC profile of the type I collagen tryptic peptides containing residue pro-986 from skin of wild type and knockout mice.
(0.62 MB TIF)
