# Supplementary figures and images for: Severe Osteogenesis Imperfecta in Cyclophilin B–Deficient Mice
Source: PLoS Genet. 2009 Dec 4;5(12):e1000750. doi: 10.1371/journal.pgen.1000750 (PMC2777385; doi:10.1371/journal.pgen.1000750)

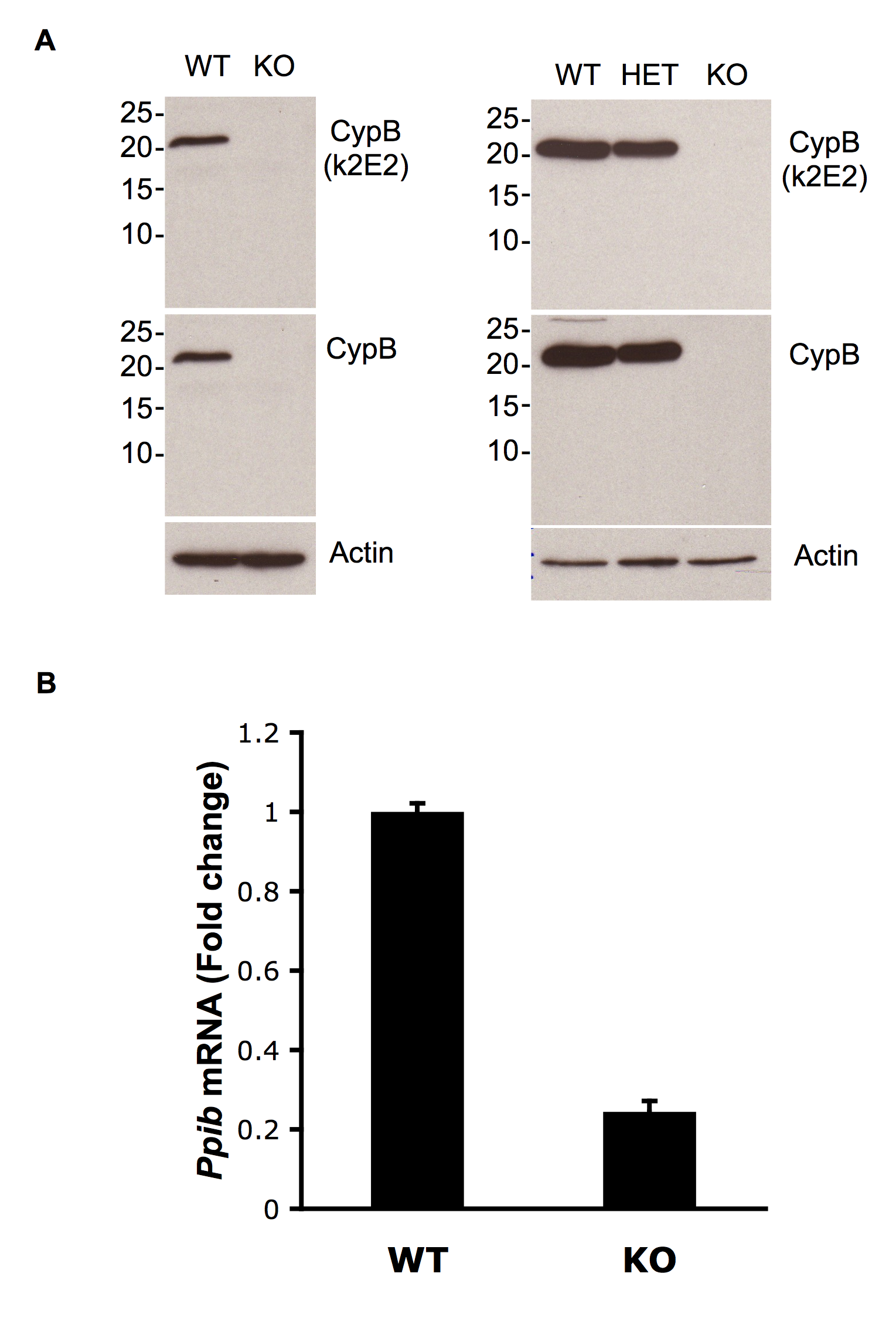

Supplement: Figure S1 — (A) Western blot of lysates from splenocytes (left) and MEFs (right) using two different antibodies to verify absence of immunoreactive CypB in knockout cells. (B) Real time rtPCR of CypB mRNA normalized to actin message levels in splenocytes from wildtype or knockout animals. (1.19 MB TIF) [file pgen.1000750.s001.tif]

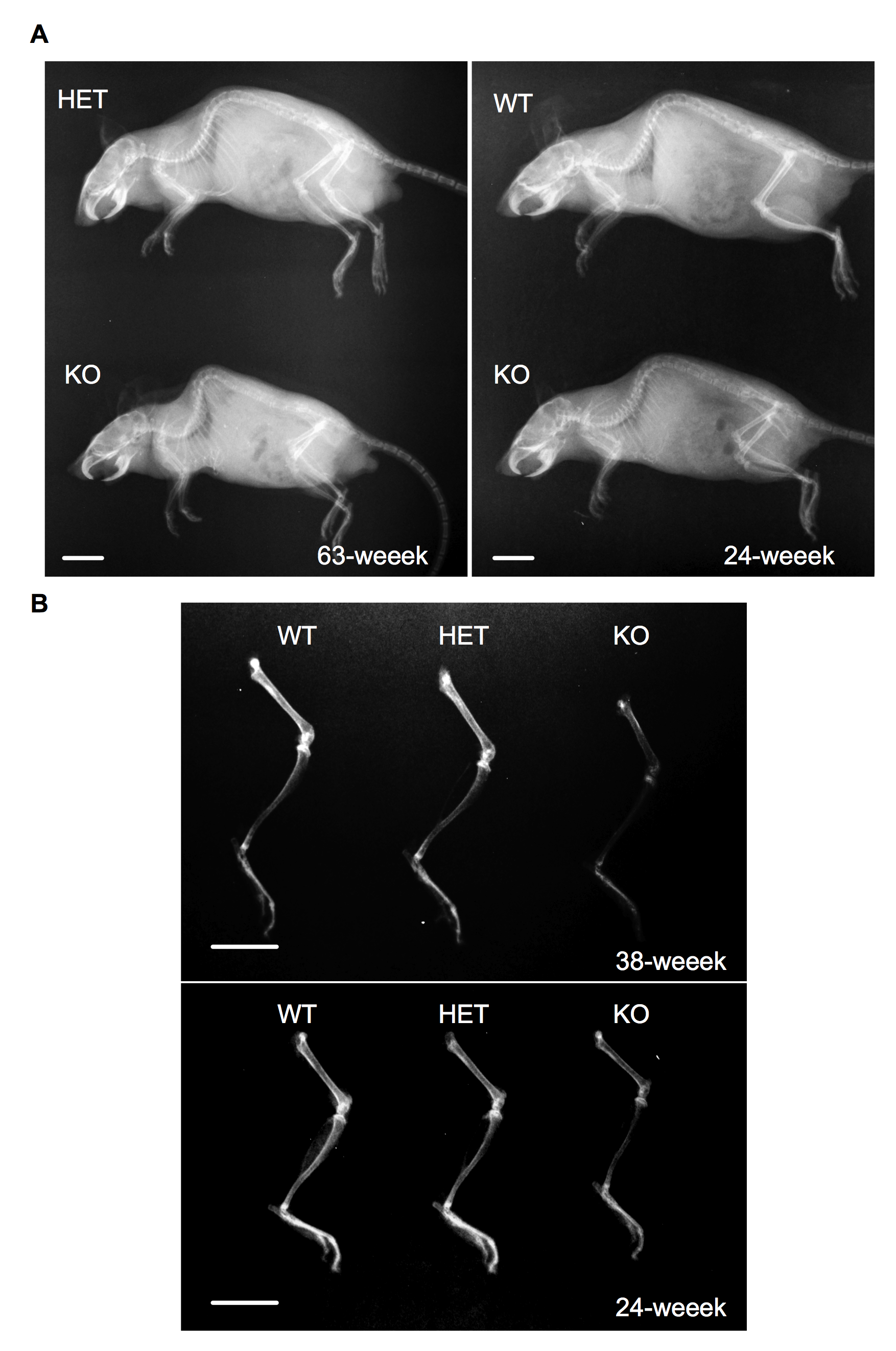

Supplement: Figure S2 — Radiographs. (A) Total body radiographs showing the skeletons of mice at 24 and 63 weeks of age. Bar = 1 cm. (B) Radiographs of lower limbs. The measured ratios of femur to tibia were similar in all mice, regardless of CypB. (1.86 MB TIF) [file pgen.1000750.s002.tif]

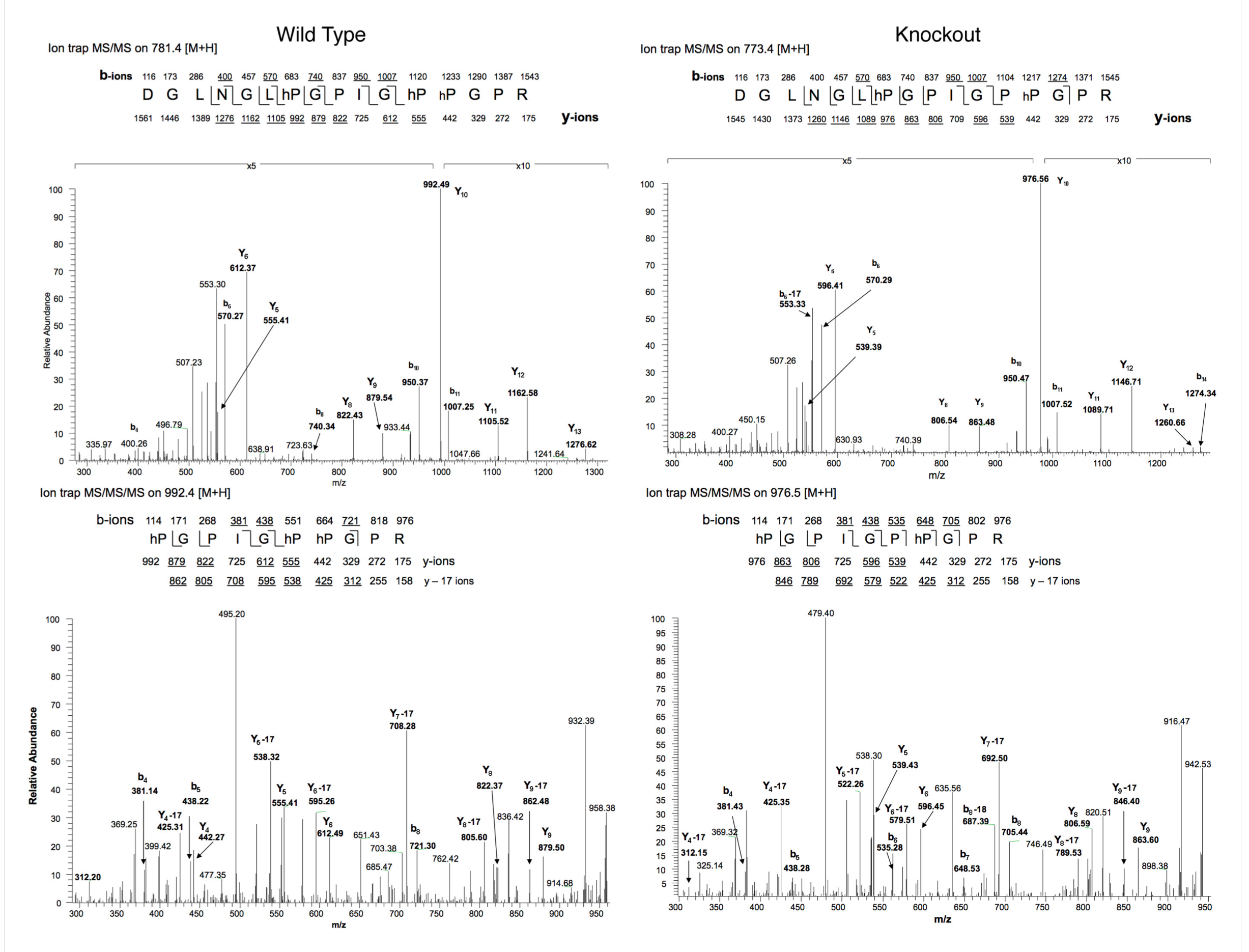

Supplement: Figure S3 — MS2 and MS3 analysis of peptides from bone, demonstrating the data used for identification of hydroxylated proline residues. (1.95 MB TIF) [file pgen.1000750.s003.tif]

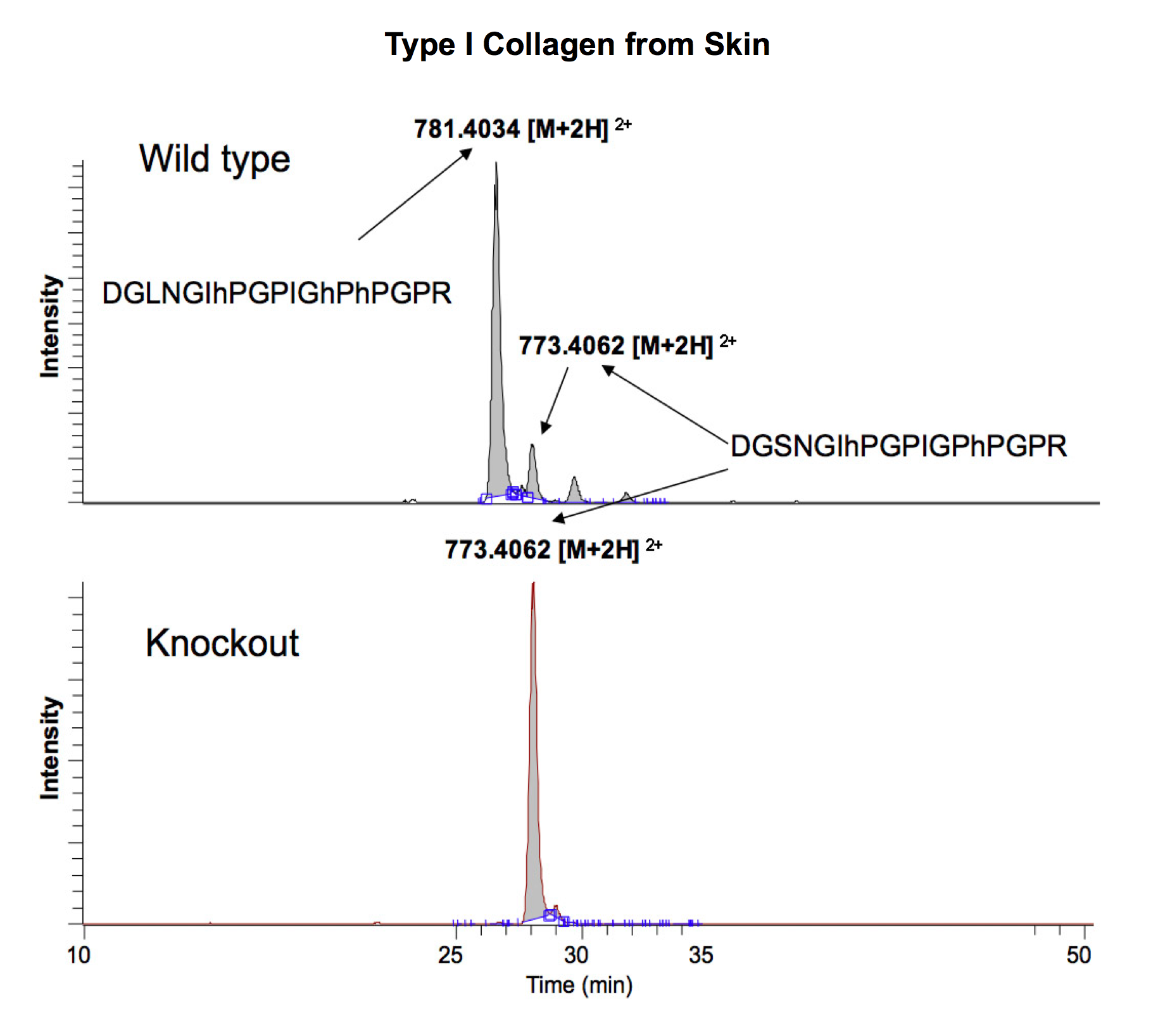

Supplement: Figure S4 — Ion-current LC profile of the type I collagen tryptic peptides containing residue pro-986 from skin of wild type and knockout mice. (0.62 MB TIF) [file pgen.1000750.s004.tif]
